# Supplementary material for: Patients with SARS-CoV-2-Induced Viral Sepsis Simultaneously Show Immune Activation, Impaired Immune Function and a Procoagulatory Disease State
Source: Vaccines (Basel). 2023 Feb 13;11(2):435. doi: 10.3390/vaccines11020435 (PMC9960366; doi:10.3390/vaccines11020435)
Supplement: Supplementary file 1 [file vaccines-11-00435-s001.zip › vaccines-2191658 supplementary.pdf]

Supplementary Material

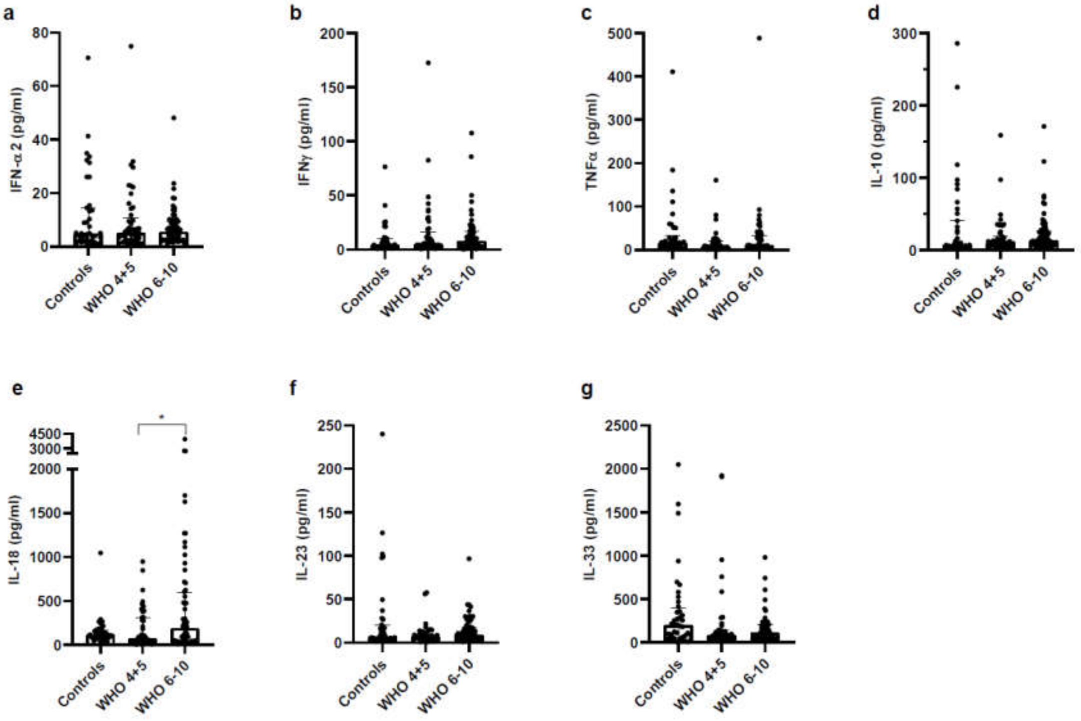

Figure S1. Additional pro-inflammatory cytokines. Platelet-free heparinized plasma of healthy controls, non-severe-COVID-19 and severe-COVID-19 patients was analysed using a LegendPlex panel and measured on a Cytoflex S). Analysis was performed according to the protocol and use of software from BioLegend. Cytokines measured were a) IFNa 2, b) IFNg, c) TNFa, d) IL-10, e) IL-18, f) IL-23, g) IL-33. Statistical comparison between groups was done by using Kruskal Wallis. All changes observed are statistically not significant.

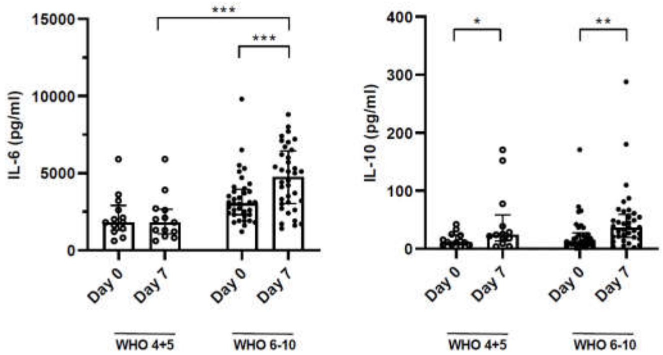

Figure S2. Time course of pro- and anti-inflammatory cytokines. Platelet-free heparinized plasma of healthy controls, non-severe-COVID-19 and severe-COVID-19 patients was analysed on day 0 and day 7 using a LegendPlex panel and measured on a Cytoflex S). Analysis was performed according to the protocol and use of software from BioLegend. Cytokines measured were a) IL-6 and b) IL-10. Statistical comparison between groups was done by using Two-way ANOVA (IL-6) and Mixed-effects analysis (IL-10). Unless marked with a p-value, all changes observed are not significant; * $p<0.05$; ** $p<0.01$; *** $p< 0.001$.
